# 7 T renal MRI: challenges and promises

**DOI:** 10.1007/s10334-016-0538-3

**Published:** 2016-03-23

**Authors:** Anneloes de Boer, Johannes M. Hoogduin, Peter J. Blankestijn, Xiufeng Li, Peter R. Luijten, Gregory J. Metzger, Alexander J. E. Raaijmakers, Lale Umutlu, Fredy Visser, Tim Leiner

**Affiliations:** Department of Radiology, University Medical Centre Utrecht, Post box 85500, 3508 GA Utrecht, The Netherlands; Department of Nephrology, University Medical Centre Utrecht, Utrecht, The Netherlands; Department of Radiology, Centre for Magnetic Resonance Research, University of Minnesota Medical School, Minneapolis, MN USA; Department of Diagnostic and Interventional Radiology and Neuroradiology, University Hospital Essen, Essen, Germany; Philips Healthcare, Best, The Netherlands

**Keywords:** 7 T MRI, Renal MRI, Kidney, RF shimming

## Abstract

The progression to 7 Tesla (7 T) magnetic resonance imaging (MRI) yields promises of substantial increase in signal-to-noise (SNR) ratio. This increase can be traded off to increase image spatial resolution or to decrease acquisition time. However, renal 7 T MRI remains challenging due to inhomogeneity of the radiofrequency field and due to specific absorption rate (SAR) constraints. A number of studies has been published in the field of renal 7 T imaging. While the focus initially was on anatomic imaging and renal MR angiography, later studies have explored renal functional imaging. Although anatomic imaging remains somewhat limited by inhomogeneous excitation and SAR constraints, functional imaging results are promising. The increased SNR at 7 T has been particularly advantageous for blood oxygen level-dependent and arterial spin labelling MRI, as well as sodium MR imaging, thanks to changes in field-strength-dependent magnetic properties. Here, we provide an overview of the currently available literature on renal 7 T MRI. In addition, we provide a brief overview of challenges and opportunities in renal 7 T MR imaging.

## Introduction

Since publication of the first in-vivo magnetic resonance imaging (MRI) study of the human brain at 8 T [1], imaging at fields equal to or higher than 7 Tesla (7 T) has been mostly limited to neuroimaging and, to a lesser degree, the extremities [2]. The first body images were presented in 2007 [3], showing the potential of prostate imaging at 7 T. Driven by the ambition to increase spatial and temporal resolution and to improve contrast, 7 T body MRI emerged further, and the first abdominal images were published in 2009 [4]. Nowadays, 7 T MRI even is used in cardiac imaging, one of the most challenging fields in MR imaging [5, 6].

The increase in signal-to-noise ratio (SNR) at higher field strengths can be used to increase spatial resolution or to decrease imaging time by using under sampling strategies such as parallel imaging or compressed sensing. In addition to the increased SNR, several other features need to be considered when imaging at high fields. First, there is the increased water-fat shift due to the higher proton Larmor frequency, which can result in larger chemical shift artefacts. However, the larger water-fat shift can potentially be utilized to improve fat suppression. The second issue that needs to be considered is the increased severity of susceptibility effects at 7 T, resulting in a decrease in *T*_2_* relaxation times [7]. Just as with the increase in water-fat shift, this has both advantages and drawbacks. In imaging techniques utilizing susceptibility differences, like blood oxygen level-dependent (BOLD) MRI or dynamic susceptibility contrast perfusion imaging, an increase in signal can be achieved. However, increased susceptibility effects may also give rise to larger geometric distortions and/or signal loss. Third, relaxation times change at increasing field strengths. In the kidney, this results in an increase in *T*_1_ relaxation time and to a lesser degree a decrease in *T*_2_ relaxation times [8]. For standard paramagnetic contrast agents, the *R*_1_ relaxivity remains similar or decreases slightly [9–11] while *R*_2_* relaxivity increases dramatically in inhomogeneous compartments such as blood [10]. To date, standard recommended doses of contrast have been administered at 7 T for providing the desired *T*_1_ enhancement, however, optimal dosing has not yet been investigated. Taking advantage of the increased *T*_1_ at 7 T along with attempts to minimize *R*_2_* effects allow for improved performance when using lower doses while simultaneously addressing concerns related to long-term effects of contrast agent administration. The increase in *T*_2_* signal attenuation is also of particular interest in dynamic contrast-enhanced (DCE) MRI studies, where contrast agent concentrations are calculated for the quantification of pharmacokinetic parameters or flow. Without correction for *T*_2_* weighting, the increased *T*_2_* weighting could introduce large errors in quantitative results [10]. Fourth, the increase in Larmor frequency at high field strengths implies a decrease in radiofrequency (RF) wavelength, which is about 12 cm in tissue at 7 T—shorter than the typical diameter of the human torso. This results in RF interference patterns creating inhomogeneous excitation and possibly areas devoid of signals where interference is destructive [4]. Fifth, the global specific absorption rate (SAR), a measure for energy absorbed by the body per unit of time, increases with the square of the electric component of the electromagnetic field [12]. Since SAR is limited to maintain temperature rises in the body below 1 °C, this imposes restrictions on the sequences that can be used [12]. At 7 T, the large RF inhomogeneity results in potential increases in local SAR, and even more importantly in local heating, which significantly impacts the types of acquisition methods, RF pulses, and sequence timings that can be used [13, 14].

Multiple groups have worked on these opportunities and challenges, and 7 T MRI is increasingly used for abdominal MRI, including renal imaging. This review aims to provide an overview of the work currently published on renal 7 T MRI in humans from the perspective of relevant renal anatomy, physiology, and current clinical practice. In the second part, challenges in renal imaging at 7 T will be addressed and a short overview of possible solutions will be provided.

## Anatomy and physiology of the kidney

**Fig. 1:**
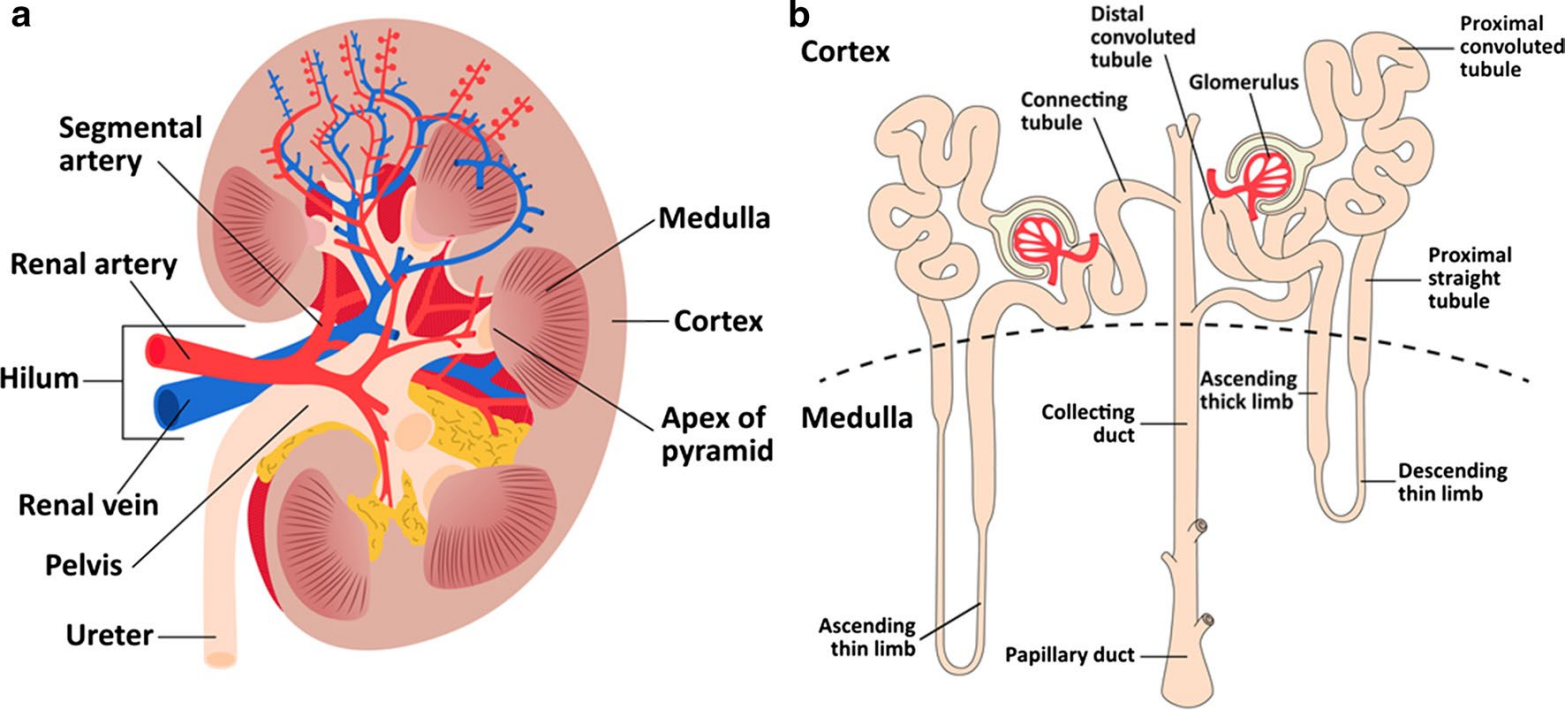
**a** Anatomy of the kidney and **b** the nephron. In the glomerulus, the blood is filtered. In the proximal convoluted tubule, most of the filtrate—both water and ions—is reabsorbed. The loop of Henle (consisting of the proximal straight tubule, the descending thin limb, and ascending thin and thick limb) concentrates the preurine and in the distal convoluted tubule more NaCl is reabsorbed [16]

The kidneys measure approximately 10–11 cm in the craniocaudal direction and about 4 cm in the anteroposterior direction, but renal size strongly depends on body size [15, 16]. They consist of an inner medullary part and a superficial renal cortex [15], with a thickness of about 1 cm [17] (Fig. 1a). The renal medulla itself consists of the renal pyramids, the apices of which point towards the renal hilum [15]. The pyramids contain tubuli, which empty into the calyces surrounding the apices of the pyramids. The calyces merge into the renal pelvis, from which the ureter originates. Through the hilum run the ureter and the renal artery and vein. The functional unit of the kidney is the nephron, which itself consists of a glomerulus, the proximal convoluted tubule, the loop of Henle and the distal convoluted tubule (Fig. 1b). Ultimately, collecting ducts lead the urine to the renal pelvis [18].

The renal artery enters the renal hilum between the renal pelvis and the renal vein, the renal pelvis lying posteriorly [19]. Thereafter, the artery divides in multiple branches: usually one posterior and four anterior segmental arteries. Branches of the segmental renal arteries run in between the medullary pyramids to the cortex. There, they give rise to the afferent arteries supplying the glomeruli [18]. The efferent arteries leaving the glomeruli supply two vascular networks: cortical peritubular capillaries and the vasa recta which descend into the medulla. The descending and ascending vasa recta are closely bundled, which allows the exchange of solutes—for example, oxygen.

Renal filtration occurs in the glomeruli [18]. The filtrate flows through the tubuli, where ions, e.g., sodium, are reabsorbed and other substances are secreted into the urine. Due to the renal concentrating mechanism, solutes—mostly sodium and chloride ions—are trapped inside the medulla, resulting in an increase in sodium concentration towards the medulla. Changes in renal tissue sodium concentration potentially yield valuable information on renal function [20].

Sodium reabsorption occurs in almost all parts of the nephron, but mainly in the medulla [18, 21]. Here, in the thick ascending limb of Henle’s loop, it is an active process, which largely accounts for the high medullary oxygen demand. However, oxygen delivery is limited in the medulla, both due to limited perfusion (medullary perfusion is about one-fifth of cortical perfusion) and due to the abovementioned counter-current exchange of oxygen in favour of the ascending vasa recta. This leaves the medulla in hypoxic conditions, susceptible to hypoxic damage [21]. Medullary oxygenation can be increased by pharmacological inhibition of oxygen-demanding processes such as sodium reabsorption. An example is furosemide, which inhibits the reabsorption of sodium in the thick ascending limb of the loop of Henle, an effect that can be measured with BOLD MRI [22].

## Clinical practice and potential of renal functional MRI

Currently, diagnostic possibilities in nephrology are limited. Creatinine clearance is the most commonly used test to assess renal function. However, at best this offers a rough estimate of glomerular filtration rate (GFR) of both kidneys combined. Comparison of plasma and urine osmolality provides some information on renal concentrating capability. In addition, the presence of erythrocytes, protein, and glucose in urine provide information on renal pathology. To obtain detailed information on renal disease, a renal biopsy is required. However, this invasive procedure occasionally leads to major haemorrhagic complications ranging from transient haematuria, occurring in 1–10 % of the cases, to requirement of blood transfusion or surgery in 0.3–7.4 and 0.2–0.5 % of the cases, respectively [23]. Imaging is used when renal artery stenosis or renal masses are suspected, and is limited to anatomical imaging or angiography [24, 25]. Functional renal MRI offers the opportunity of expanding the diagnostic possibilities in renal disease, and potentially may decrease the need for renal biopsy.

Currently, functional MRI of the kidneys is being investigated in a range of diseases, including renal tumours [26], renal transplantation [27], renal artery stenosis [28, 29], acute kidney injury [30], and chronic kidney disease [31, 32]. DCE MRI can potentially be utilized to measure single kidney glomerular filtration rate, although application is still limited due to challenges in post-processing of data, lack of standardized protocols, and fear of development of nephrogenic systemic fibrosis due to contrast agents [33].

In the assessment of radiologically indeterminate renal masses, the European Association of Urology advises clinicians to perform renal biopsy, although “advanced MRI techniques such as diffusion-weighted and perfusion-weighted imaging are being explored for renal mass assessment” [25]. This exploration is in an advanced phase: a recent review states that a multiparametric MRI protocol combining anatomical sequences with chemical shift, diffusion-weighted and DCE imaging “is becoming the reference standard for renal mass imaging in clinical practice” [26]. In other renal diseases, functional MRI is still under investigation. In renal artery stenosis, renal BOLD and DCE MRI potentially can be used to identify patients that are likely to benefit from revascularization [28, 29]. Also, the measurement of physiological parameters in chronic kidney disease can be of interest. In this condition, kidney ischemia is thought to initiate a cascade of physiological changes, including activation of the sympathetic nervous system and the renin-angiotensin-aldosterone system, ultimately resulting in an increase in blood pressure and a decrease in GFR [34]. This mechanism is also thought to play an important part in the development of hypertension. However, in a large study no correlation between renal *T*_2_* and GFR was found [31]. This might be explained by the delicate balance of oxygen consumption and delivery described earlier and the fact that *T*_2_* is related to blood oxygenation, contrary to tissue oxygenation [35]. Therefore, *T*_2_* measurements should be combined with measurements of renal blood flow in the assessment of renal oxygenation [35]. Also, measurements of renal sodium concentration could provide valuable information in chronic kidney disease, since both the renin-angiotensin-aldosterone system and the sympathetic nervous system influence renal sodium handling [34]. As will be shown below, benefits of 7 T are most distinct for functional imaging techniques such as BOLD MRI, arterial spin labelling (ASL), and sodium imaging.

## Currently published work on renal 7 T MRI

### Anatomical imaging

**Fig. 2:**
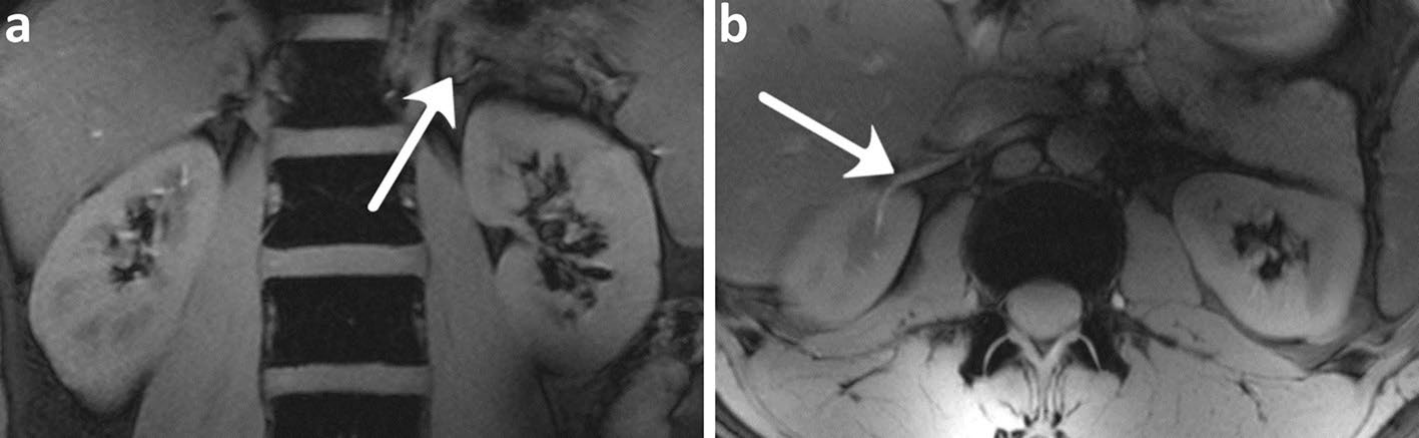
Coronal *T*_1_w 2D FLASH images: **a** *arrow* adrenal gland **b** *arrow* renal vasculature with high signal intensity (Umutlu et al. unpublished results)

**Fig. 3:**
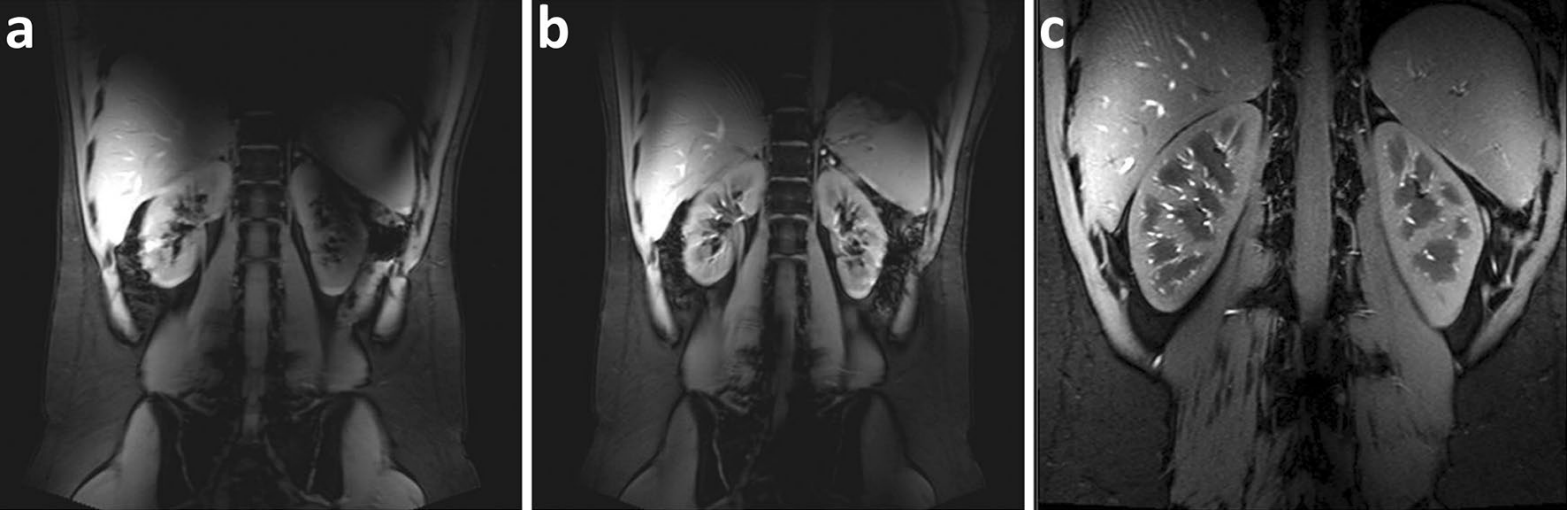
Coronal *T*_1_w gradient echo images **a** without B_1_^+^ shimming, **b** with local B_1_^+^ shimming and **c** a high-resolution version of **b** (FOV 240 mm, slice thickness 2.2 mm, remaining parameters the same) (Metzger et al., reprinted with permission from [14])

**Fig. 4:**
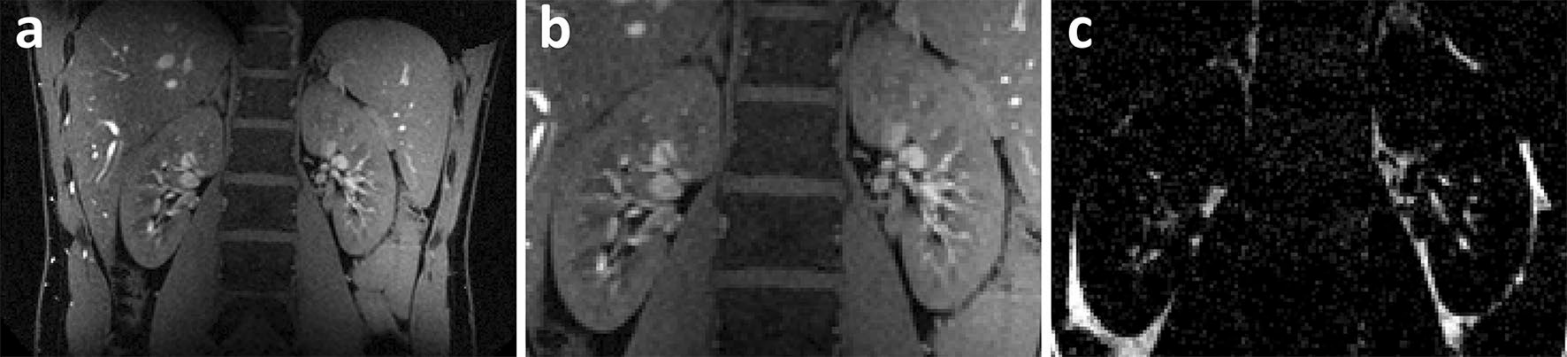
Coronal TFE images with Dixon reconstruction; **a** water image, **b** zoom of **a**, and **c** fat image (Hoogduin et al. [37])

The first to investigate feasibility of renal MRI at 7 T were Umutlu et al. [36]. Of the *T*_1_-weighted sequences used, 2D spoiled gradient echo (2D FLASH, Fig. 2) and 2D in- and opposed phase gradient echo performed best in depicting the renal structures. Other *T*_1_-weighted images showing better contrast between cortex and medulla were acquired by Metzger et al. [14] (Fig. 3). Hoogduin et al. [37] have aimed to extend the work of Umutlu [36] and Metzger et al. [14] by using a multi-echo turbo field echo sequence with Dixon reconstruction for fat suppression (Fig. 4). Initial results indicate that good fat suppression can be obtained, although contrast between the medulla and cortex is somewhat less than in the images acquired by Metzger et al. As clearly seen in Figs. 2, 3 and 4, arteries were hyperintense on *T*_1_-weighted sequences. This can be explained by inflow effects due to the absence of blood saturation entering from regions outside the excitation area [38].

**Fig. 5:**
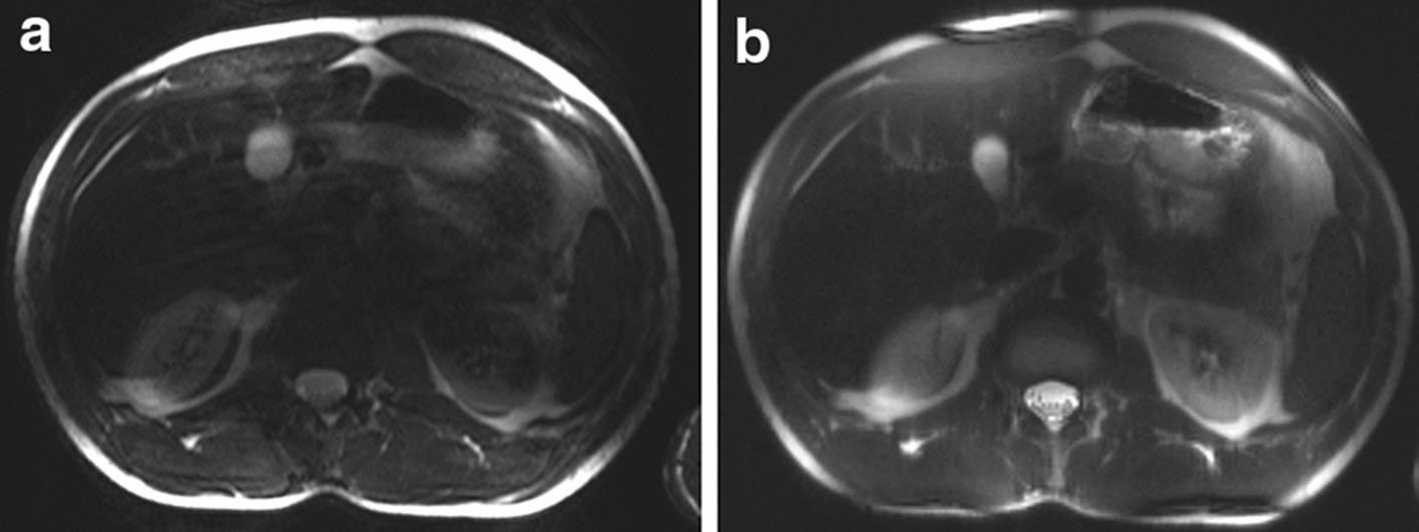
Axial *T*_2_w TSE images acquired in two subjects: **a** strongly impaired and **b** with medium image quality (Umutlu et al. unpublished results)

**Fig. 6:**
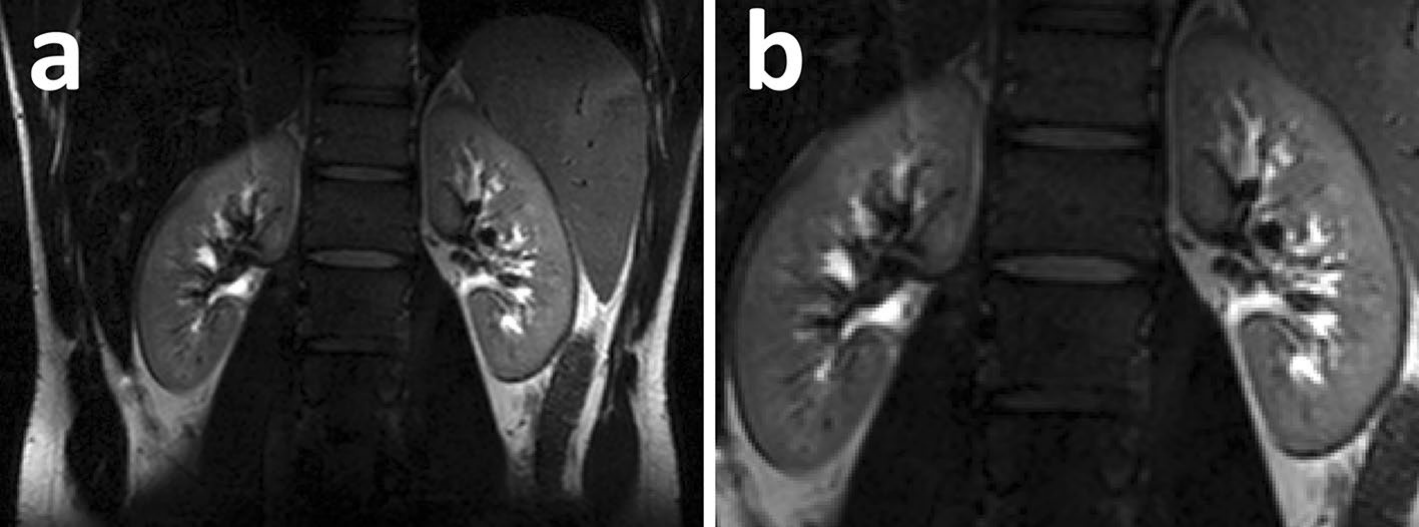
**a** Coronal *T*_2_w TSE images; **b** zoom of **a** (Hoogduin et al. [37])

Umutlu et al. [36] performed *T*_2_-weighted imaging, but the images were heavily impaired by artefacts due to B_1_ field inhomogeneity and SAR limitations (Fig. 5). *T*_2_-weighted imaging also was performed by Hoogduin et al. [37] using a turbo spin-echo (TSE) sequence, yielding good quality images with minor artefacts (Fig. 6). To keep the SAR within limits, a long TR (16,000 ms) and SENSE factor of five had to be used. In Tables 1 and 2, the parameters of the *T*_1_ and *T*_2_-weighted sequences are provided, respectively.

**Table 1:** Imaging parameters for the *T*_1_-weighted anatomical sequences

Sequence	2D FLASH [36, 39]	3D FLASH [36, 39]	*T*_1_w GE [14]	*T*_1_w ME TFE [37]
Slice orientation	Coronal	Coronal	Coronal and axial	Coronal
TR/TE (ms)	130/3.57	2.9/1.02	150/3.7	5.0/2.1;2.7;3.3
Nominal flip angle (°)	70	10	60	10
FOV (mm)	400	400	450	370
Voxel volume (mm)	0.8 × 0.8 × 2.0	1.3 × 1.3 × 1.6	1.9 × 1.2 × 5	1.49 × 1.49 × 3.00
Slices	13	128	14	12
Acquisition time (s)	31^a^	27^a,b^	18^a^	44^c^
Parallel imaging	GRAPPA 2	GRAPPA 2	GRAPPA 2	SENSE 2

*ME* multi-echo, *TFE* turbo field echo

^a^Equals breath hold length

^b^Using 66 % over contiguous slices and 6/8 partial Fourier in phase and slice direction

^c^One slice per breath hold (3.6 s)

**Table 2:** Imaging parameters for the *T*_2_-weighted anatomical sequences

Sequence	*T*_2_w TSE [36, 39]	*T*_2_w TSE [37]
Slice orientation	Axial	Coronal
TR/TE (ms)	3060/99	16,000/187
Nominal flip angle (°)	120	125
FOV (mm)	350 × 240	375 × 518
Voxel volume (mm)	1.4 × 1.4 × 5.5	1.4 × 1.6 × 5.0
Slices	16	5
Acquisition time (s)	34^a^	80^b^
Parallel imaging	GRAPPA 2	SENSE 5

^a^Equals breath hold time

^b^Respiratory triggering

The effect of contrast administration both on renal vasculature and corticomedullary differentiation was investigated by Umutlu et al. [39]. They acquired 3D FLASH images before and 20, 70, and 120 s after contrast administration. Contrast administered in a dose of 0.1 mmol/kg resulted in a slight improvement in contrast between the cortex and medulla and a better depiction of the renal arteries on 3D FLASH images with respect to 2D FLASH, but only in the image acquired during the arterial phase (20 s post-contrast).

### *T*_1_ and *T*_2_ measurements

**Fig. 7:**
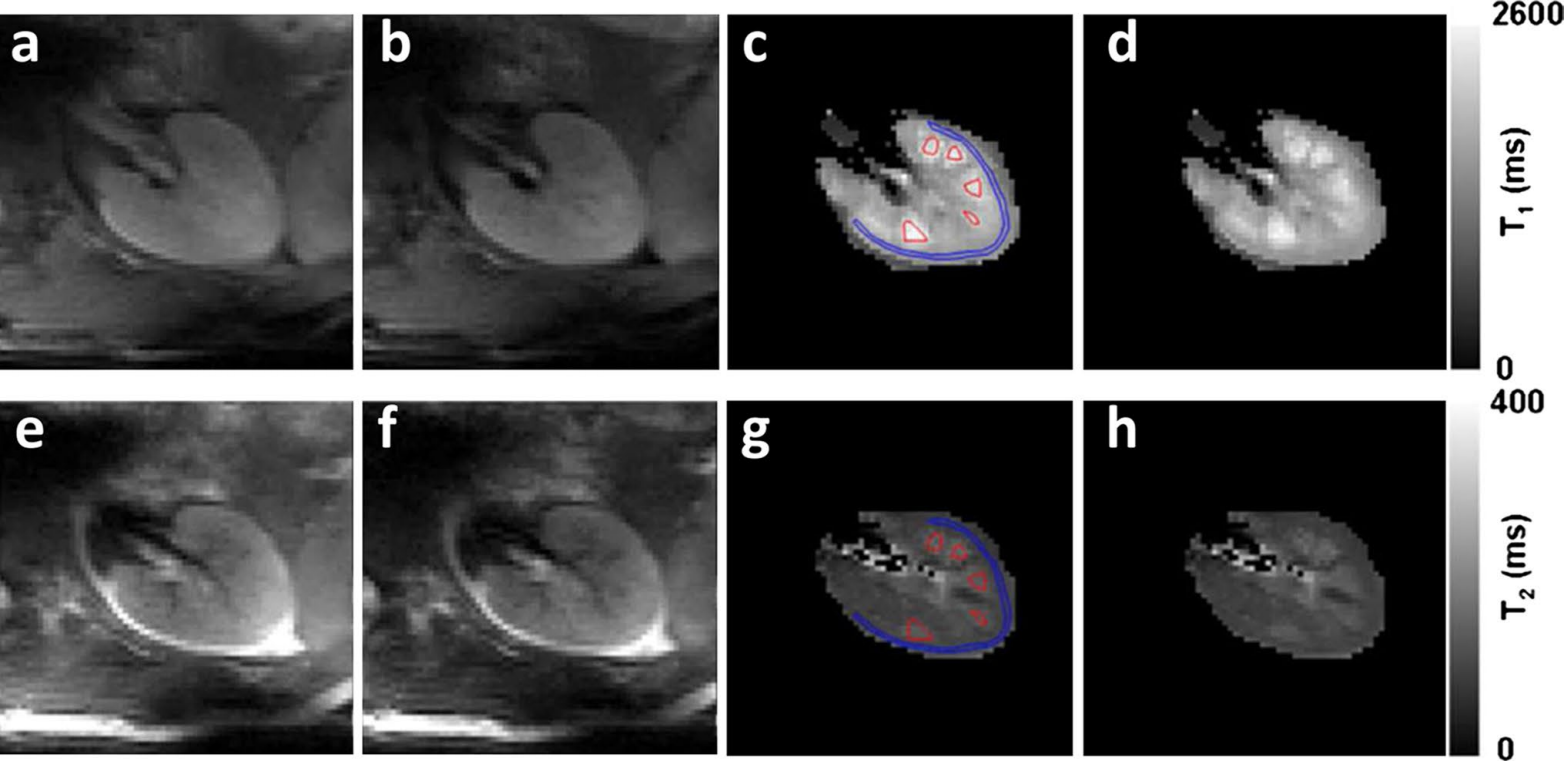
**a**, **b** ss-FSE images for *T*_1_ measurement with inversion time 100 and 150 ms, respectively; **c**, **d** *T*_1_ maps with and without ROIs for *T*_1_ estimation; **e**, **f** ss-FSE images for *T*_2_ measurement with effective echo time 20 and 40 ms; **g**, **h** *T*_1_ maps with and without ROIs for *T*_1_ estimation (all ss-FSE images were acquired at six different inversion times or effective echo times to minimize short-term SAR) (Li et al., reprinted with permission from [8])

Since proton relaxation times depend on field strength, measurements of kidney *T*_1_ and *T*_2_ are desirable to optimize scan protocols. Thus far, only one group performed measurements of relaxation times [8]. Measurements were performed in eleven healthy volunteers and five subjects were scanned twice to evaluate reproducibility. Values were compared to measurements on 3 T using the same sequences with similar parameters. To assure a uniform inversion, B_1_^+^ shimming was applied only for one kidney, resulting in a coefficient of variation of B_1_^+^ inhomogeneity of 8 %. For *T*_1_ measurements, a single-shot fast spin-echo (ss-FSE) was used with adiabatic inversion pulses at six different inversion times. For *T*_2_ measurement, a Carr-Purcell-Meiboom-Gill (CPMG) refocusing pulse train was inserted as a preparation module in between excitation and readout to minimize signal loss due to diffusion. Within this preparation module on each side of the refocusing pulses, spoiling gradients were applied to prevent the formation of stimulated echoes. Measurements consisting of a single transversal slide could be performed in a single breath hold. *T*_1_ and *T*_2_ maps are shown in Fig. 7 and mean relaxation times are given in Table 3. *T*_2_ values measured for comparison at 3 T were significantly higher than values known in the literature, possibly due to the pulse sequence, which was designed to reduce signal loss due to diffusion by insertion of a CPMG refocusing pulse train.

**Table 3:** *T*_1_ and *T*_2_ values on 3 and 7 T [8]

	Cortex		Medulla	
	*T*_1_	*T*_2_	*T*_1_	*T*_2_
3T	1261 ± 86	121 ± 5	1676 ± 94	138 ± 7
7 T	1668 ± 46	109 ± 6	2095 ± 52	125 ± 5

### Angiography

**Fig. 8:**
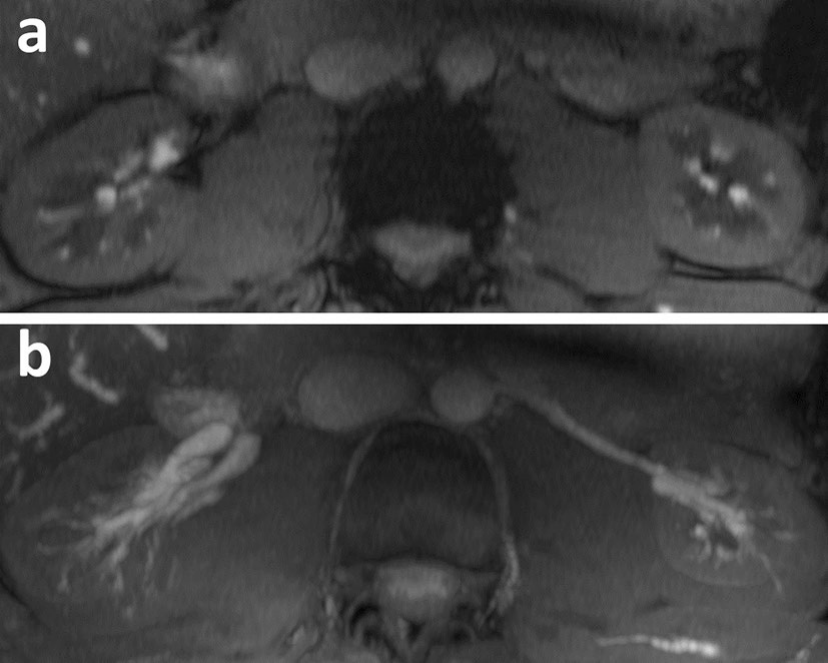
**a** TOF MRA and **b** maximum intensity projection of TOF MRA (Umutlu et al. unpublished results)

In two separate publications, Umutlu et al. described the possibility of 7 T renal MR angiography (MRA) using both native 2D gradient echo time-of-flight (TOF) MRA as well as using contrast-enhanced techniques [40, 41]. In both studies, image quality of angiographic sequences was assessed on a 5-point scale, with 1 denoting non-diagnostic and 5 denoting excellent delineation. To image renal vasculature, gradient echo TOF MRA was compared to *T*_1_-weighted 2D and 3D FLASH, where the vasculature was hyperintense as observed earlier [40]. While 2D FLASH suffered from artefacts (mainly inflow effects and motion) and low SNR and CNR, both 3D FLASH and TOF MRA performed well. In 3D FLASH, moderate background suppression led to a low contrast-to-noise ratio (CNR) with respect to the psoas major muscle, despite good SNR. The authors concluded that TOF MRA was capable of superior delineation of the aorta and left and right renal arteries compared to both 2D and 3D FLASH and superior CNR (Fig. 8), although some venous overlay was present due to insufficient background suppression. For TOF MRA, mean SNR was 54 and mean quality score was 4.7 out of 5.

**Fig. 9:**
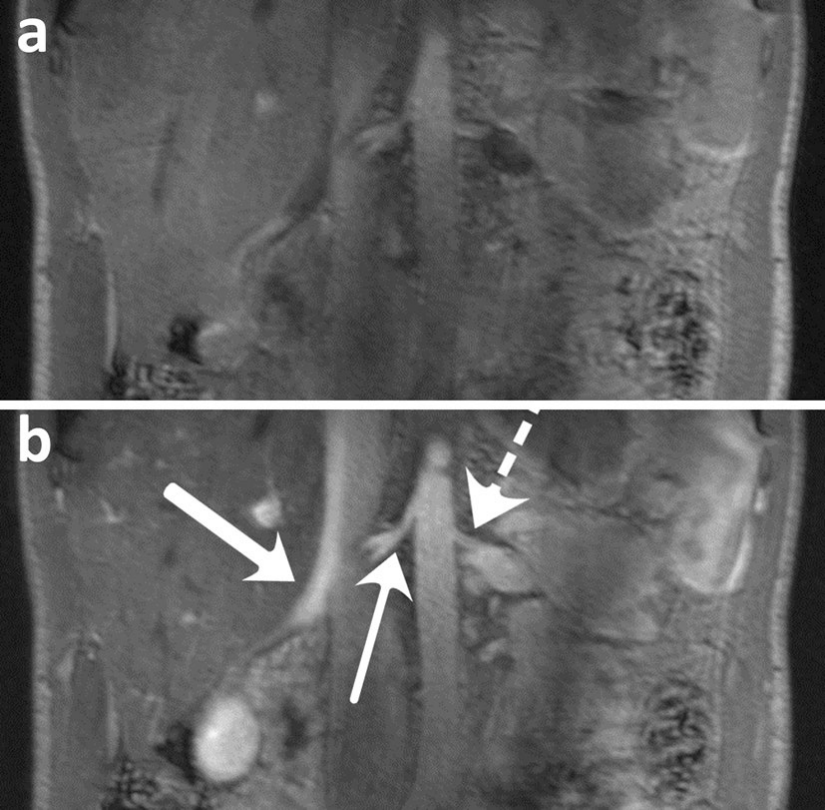
**a** Unenhanced and **b** CE 3D FLASH. *Wide arrow* right renal vein, *slim arrow* right renal artery, *dashed arrow* left renal artery (Umutlu et al. unpublished results)

Umutlu et al. also compared contrast-enhanced (CE) MRA to unenhanced 3D FLASH MRI [41]. Contrast enhancement mainly improved delineation of the right renal artery (Fig. 9), while improvement was less pronounced in the left renal artery and the aorta. CNR improved about two-fold for all arteries, ranging from 45 to 60, with SNR ranging from 90 to 105. Mean quality score of CE 3D FLASH was 4.8. However, measurements were only performed in healthy volunteers, so no information is available on the grading of renal artery stenosis on 7 T.

**Fig. 10:**
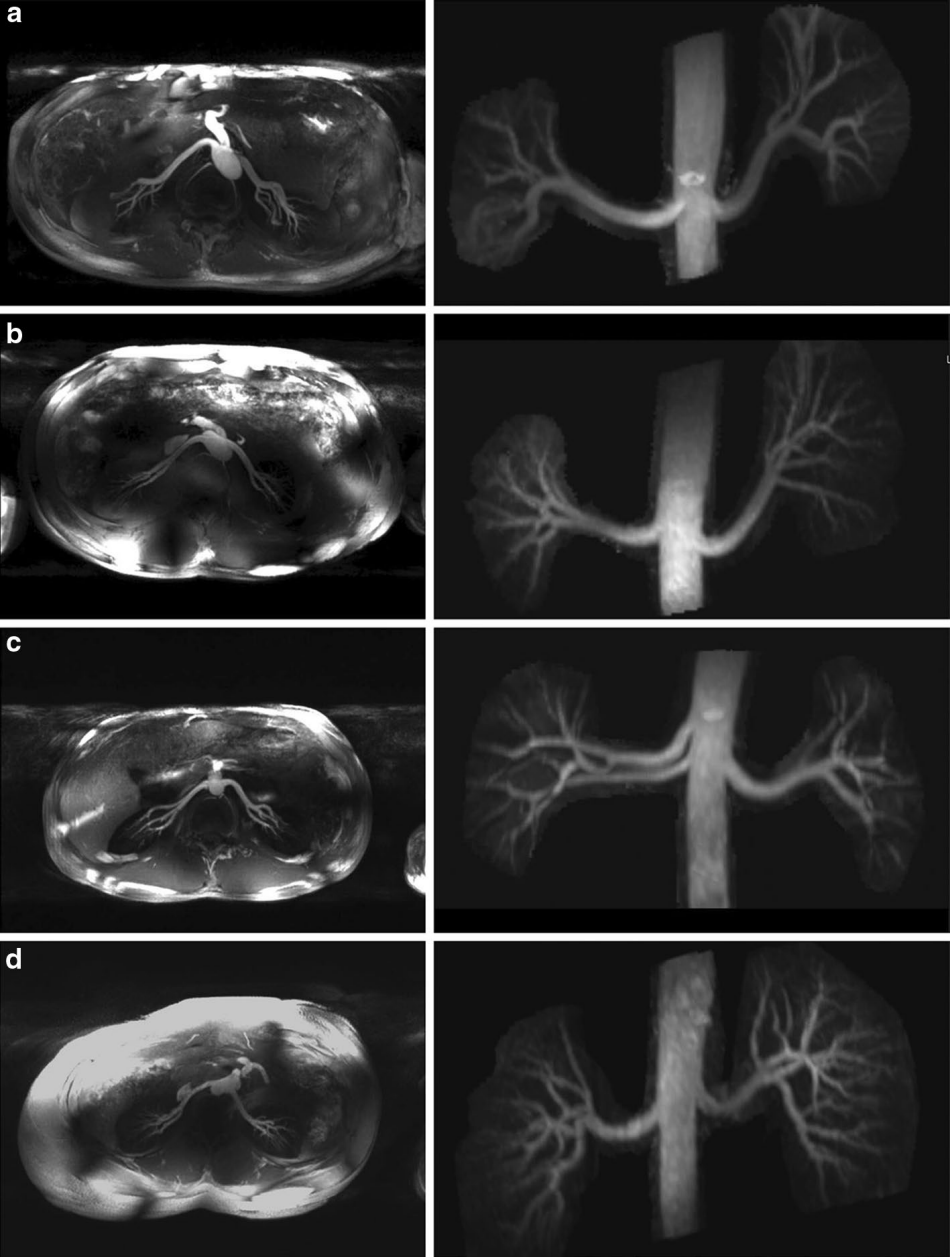
Axial (*left*) and cropped coronal (*right*) MIPs from multiple volunteers with different shimming strategies: **a** phase-only homogeneity shim both for saturation and conventional pulses; **b** efficiency shim for saturation pulse; **c**, **d** trade-off solution for saturation pulse, magnitude, and phase homogeneous shim for conventional pulses (Metzger et al., reprinted with permission from [14])

Metzger et al. also explored the possibility of renal artery imaging at 7 T using non-CE MRA with a 3D turbo FLASH sequence [14]. To minimize background signals, a frequency selective saturation pulse was used to reduce lipid signals and a slab-selective adiabatic inversion was used to null the renal parenchyma. Different B_1_^+^ shimming solutions were used for the adiabatic inversion and the other RF pulses in the sequence (i.e., the fat saturation and excitation). The inversion pulse required a much higher B_1_^+^, and thus benefitted from an efficiency solution optimized over the kidneys while the excitation and lipid suppression benefitted from more homogeneous B_1_^+^ over a larger region of interest. Dynamically applying these shim solutions within a sequence addressed the fact there is an implicit trade-off between RF homogeneity and efficiency, preventing both conditions from being accomplished simultaneously. In Fig. 10, maximum intensity projections (MIPs) are shown in multiple subjects in which this acquisition strategy was employed. Note the excellent depiction of small vessels, which are not obscured by the renal parenchyma as is often the case when using intravenously injected contrast agents. Imaging parameters for all sequences are given in Table 4.

**Table 4:** Imaging parameters for MR angiography sequences

Sequence	CE 3D FLASH [41]	2D GE TOF [40]	3D Turbo FLASH [14]
Slice orientation	Coronal	Transverse	Coronal and axial
TR/TE (ms)	2.98/0.97	17/4.70	3.8/1.76
Nominal flip angle (°)	25	60	8
FOV (mm)	400	250 × 188	300–380
Voxel volume (mm)	1.5 × 1.0 × 1.0	1.0 × 2.0 × 2.5	1.1–1.2 × 1.1–1.2 × 1.0–1.3
Slices	NR	20	72
Acquisition time	20 s^a^	33 s^a^	9 min^b^
Parallel imaging	GRAPPA 2	GRAPPA 2	GRAPPA 2
Saturation	Fat saturation	Fat saturation	Fat saturation and adiabatic inversion

*GE* gradient echo, *NR* not reported

^a^Equals breath-hold length

^b^Respiratory triggering

## Functional imaging

### BOLD MRI

**Fig. 11:**
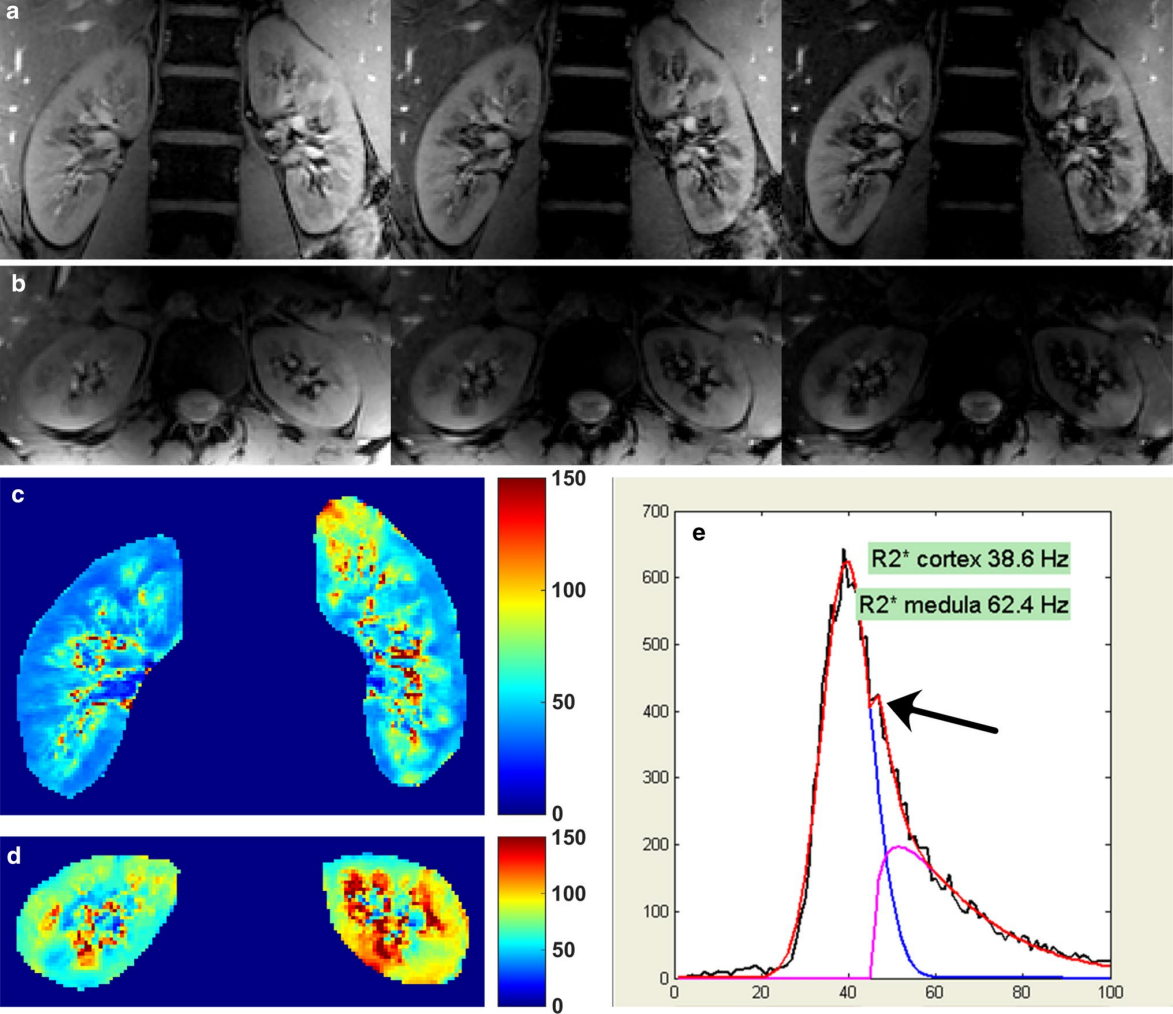
*T*_2_*-weighted images: **a** coronal images (echo times 4.9, 9.9 and 14.8 ms); **b** transversal image (echo times 4.9, 9.9 and 14.8 ms); **c**, **d** corresponding *R*_2_* maps; **e** histogram of the *R*_2_* values. In the compartmental method, the sum of a Gaussian function (*red*) representing the cortex and a gamma function (*purple*) for the medullary values is fitted to the histogram. *Arrow* distinct peak of medullary voxels, not visible on 3T data (Hoogduin et al. [37])

BOLD MRI relies on the difference between deoxygenated and oxygenated haemoglobin, the first being paramagnetic while the second is diamagnetic. Three groups performed renal BOLD MRI at 7 T. Brinkmann et al. [42] performed BOLD imaging using a multi-echo 2D FLASH sequence during water loading both on 3 and 7 T. The decrease in medullary *R*_2_* value during water loading (23 % at 3T) was more pronounced on 7 T, where a decrease of 33 % was measured. In addition, the *R*_2_* values were higher at 7 T: 90 versus 29 Hz on 3T in the medulla and 69 versus 25 Hz for renal cortex. Hoogduin et al. found similar results [37] using a multi-echo gradient echo sequence (Fig. 11). To derive *R*_2_* values for cortex and medulla, they used a compartmental analysis as proposed by Ebrahimi et al. [43]. Here, a Gaussian function representing the cortex and a gamma function for the medulla are fitted to the histogram of the data (Fig. 11e). In comparison with data measured earlier at 3T in the same centre [44], at 7 T a distinct peak for the medulla was visible on the histogram (Fig. 11e). Mean *R*_2_* values of 66 and 41 Hz for the medulla and cortex were measured, respectively. While the *R*_2_* ratio between the medulla and cortex in the study by Hoogduin et al. was nearly constant, 1.54 on 3T versus 1.60 on 7 T, Brinkmann et al. measured slightly different ratios: 1.16 on 3T versus 1.30 on 7 T. Li et al. also performed *T*_2_* mapping, but they did not publish quantitative results [8]. Scan parameters are provided in Table 5.

**Table 5:** Imaging parameters for BOLD MRI sequences

Sequence	ME 2D FLASH [42]	ME GE [37]
Slice orientation	Coronal	Coronal
TR (ms)	96	102
Number of echoes	10	20
TE (ms)	2; 6.6; …; 29.1	4.93; 9.86; …; 98.6
FOV (mm)	256	375 × 381
Voxel volume (mm)	1.0 × 1.0 × 6	1.5 × 1.5 × 5
Slices	3	3
Acquisition time (s)	24	47^a^
Parallel imaging	NR	SENSE 2

*NR* not reported, *ME* multi-echo

^a^One slice per breath hold (16 s)

### Arterial spin labelling

**Fig. 12:**
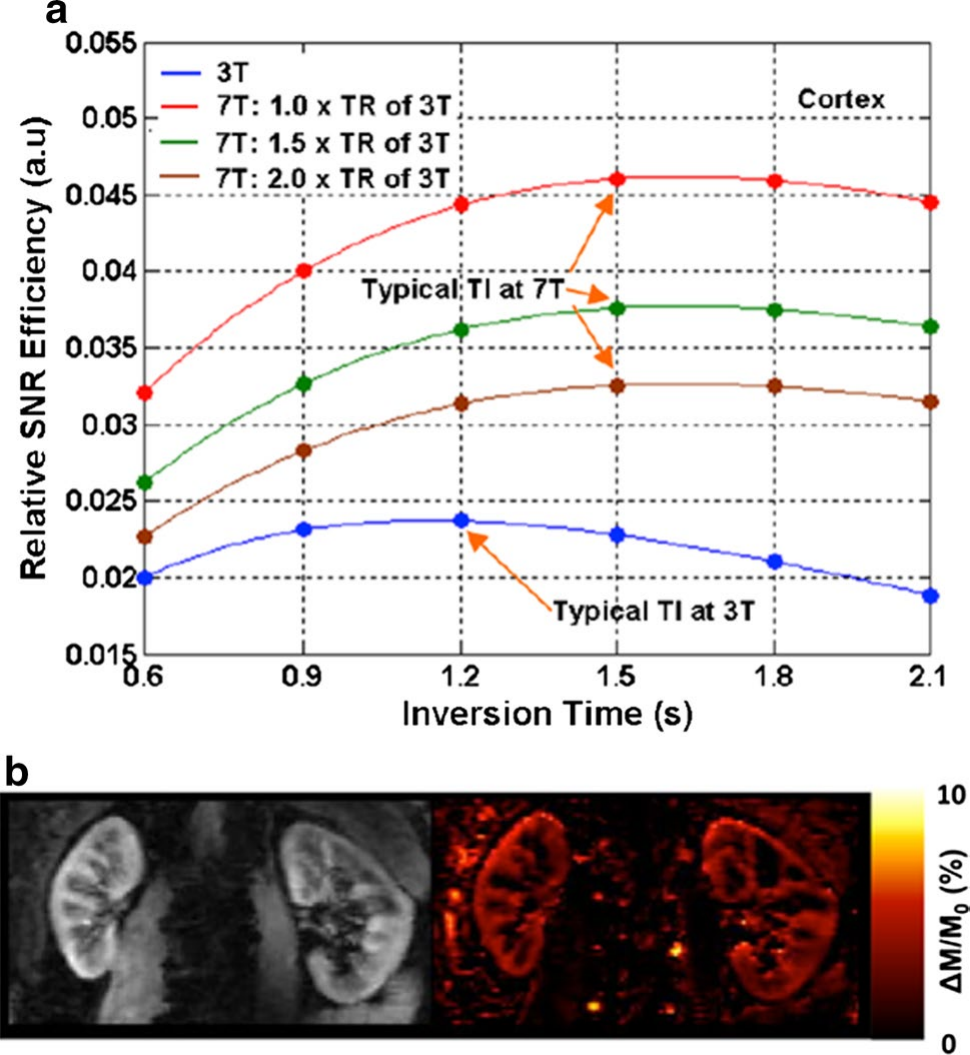
**a** Theoretical simulations of renal perfusion SNR efficiencies at 3 T and 7 T for renal perfusion imaging using FAIR EPI. TR represents repetition time. **b** One subject’s proton (*left*) and normalized perfusion-weighted (*right*) images from perfusion study using FAIR-EPI at 7 T with 2 × 2 × 5 mm^3^ resolution. ΔM represents perfusion-weighted signal evaluated as the signal difference between label and control images, and *M*_0_ the fully relaxed renal tissue signal (Li et al. [48])

Non-contrast-enhanced renal perfusion imaging using ASL is an attractive approach for studying renal physiology and assessing renal diseases, and is well-suited for the longitudinal monitoring of renal function. In ASL MRI, the arterial blood is used as an endogenous tracer [45] by labelling it either with an adiabatic inversion RF pulse as in flow-sensitive alternating inversion recovery (FAIR) or a pseudo-continuous RF pulse train as in pseudo-continuous spin labelling (PCASL). Unfortunately, the intrinsically low SNR of ASL requires a large number of signal averages and correspondingly long imaging acquisition times, thus imposing critical limitations on its clinical application. After the inversion used in both FAIR and PCASL, the labelled spins that perfuse the area of interest disappear at a rate *R*_1_ = 1/*T*_1_, thus the longer *T*_1_ at 7 T increases the much needed SNR in ASL studies. It has been shown that 7 T can specifically benefit renal ASL perfusion imaging due to increased SNR, prolonged blood [46] and renal tissue [8] longitudinal relaxation times, and improved parallel imaging performance [47]. Theoretical simulations of renal perfusion SNR efficiency at 7 T and 3 T by using EPI as an imaging readout suggest that, compared to 3 T, 7 T can benefit renal ASL perfusion imaging by providing higher SNR efficiency even if longer repetition times are needed due to possible SAR constraints (Fig. 12a) [48]. Initial 7 T studies suggest that there are no SAR issues for renal perfusion imaging using FAIR EPI with respiratory triggering acquisition [49, 50]. The renal perfusion images from such a study are presented in Fig. 12b [48].

**Fig. 13:**
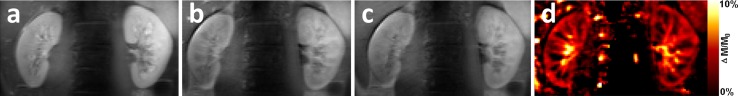
PASL images acquired with ss-FSE readout: **a** proton density; **b** control image; **c** labelling **d** perfusion-weighted imaging normalized to proton density (Li et al. [50])

To avoid image distortions due to the sensitivity of an EPI readout to susceptibility effects and to take advantage of the dramatically increased perfusion SNR at 7 T, the feasibility of single breath-hold renal ASL perfusion imaging was demonstrated by using a single shot-fast spin-echo (ss-FSE) as the imaging readout [50] in combination with either pulsed ASL (PASL) using FAIR or PCASL. Although it is expected that PCASL can provide a higher perfusion SNR efficiency than FAIR, the limited coverage of the transceive body arrays used in these studies greatly limits the ability to label the blood in the descending aorta above the imaging field of view. This reduces the possible gains that PCASL theoretically can provide. In addition, compared to FAIR, PCASL is a much more power-intensive method, which poses additional problems at 7 T [45]. To limit SAR, the TR had to be lengthened with this approach, resulting in an increased imaging time that made it challenging to complete the sequence within a single breath hold [50]. Also, the use of ss-FSE as readout yielded an increase in SAR compared to EPI, which necessitated the use of high parallel imaging factors and hyper-echoes for reducing the amount of time-averaged power delivered. In Fig. 13, images acquired using FAIR and an ss-FSE readout are shown.

### Sodium imaging

Haneder et al. [51] published the only work on renal sodium imaging at 7 T. Due to a low sodium concentration, very short transverse relaxation times and intrinsically low MR sensitivity of the ^23^Na nucleus, in-vivo sodium imaging is challenging. Theoretically, the ^23^Na SNR at 7 T would be 2.33 times the SNR at 3T [51]. In addition, due to the lower Larmor frequency of the ^23^Na nucleus resulting in a wavelength of about 45 cm, sodium imaging at 7 T is hardly complicated by B_1_ inhomogeneity effects.

**Fig. 14:**
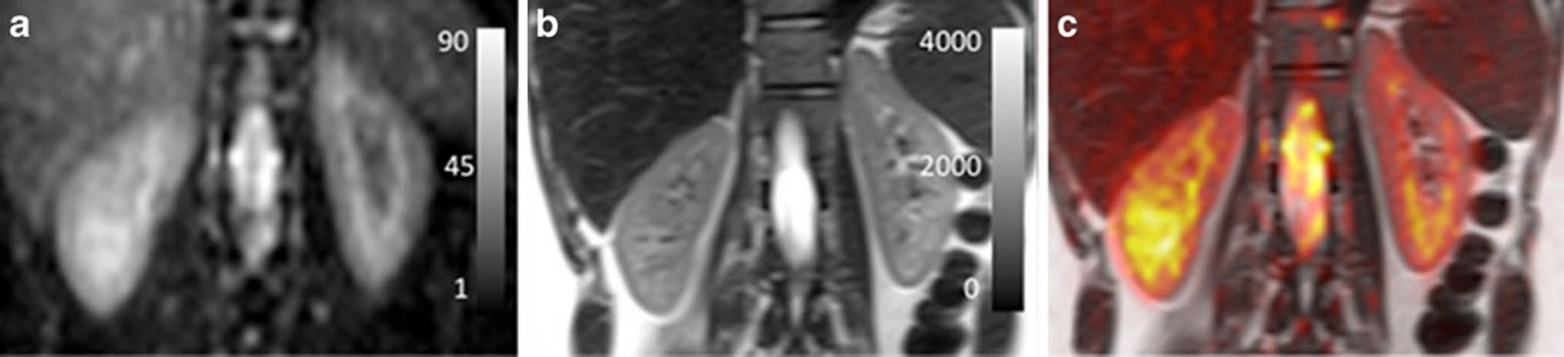
Coronal images: **a** ^23^Na image on 7 T with scale representing ^23^Na SNR in arbitrary units and **b** corresponding *T*_2_-weighted proton image in the same subject on 3 T with scale representing signal intensity in arbitrary units, **c** *T*_2_-weighted image with overlaid colour-encoded ^23^Na image (Haneder et al., reprinted with permission from [51])

The transverse relaxation of the ^23^Na nucleus can be described by a biexponential function, consisting of a fast and a slow component [20, 51]. The aim of the study was to perform the first measure of the slow ^23^Na *T*_2_* component in humans. In addition, morphological ^23^Na images were made (Fig. 14). Imaging parameters for the morphological 3D spoiled GE sequence and the multi-echo 3D variable echo time (vTE) GE sequence for *T*_2_* mapping are given in Table 6. The authors concluded that sodium imaging benefits from the increased field strength. The spatial resolution of morphological ^23^Na images could be increased to 4 × 4 mm^2^, compared to 5 × 5 mm^2^ on 3 T [52], with improved image quality compared to lower field strengths. As in earlier studies, a cortico-medullary gradient could be observed with increasing sodium concentration in the medulla. The value was comparable: 4.1 ± 0.35 mmol/l/mm compared to 3.38 ± 0.35 mmol/l/mm, as the same group measured earlier [52]. The measured slow component of *T*_2_* values of 17.9 and 20.6 ms for the cortex and medulla were in agreement with values published in animal studies on different field strengths (ranging from 11 to 36 ms on 2.1 to 17.6 T) [51].

**Table 6:** Imaging parameters for the sodium imaging sequences [51]

Sequence	3D spoiled GRE	Multi-echo 3D vTE GRE
Slice orientation	Coronal	Coronal
TR (ms)	49 (38–61)	75
TE (ms)	4.19	2.64; 4.93; 13.76; 19.18; 24.59; 30.01; 40.00; 45.42; 55.00; 60.42
Nominal flip angle (°)	NR	NR
FOV (mm)	256	256
Voxel volume (mm)	4 × 4 × 5	4 × 4 × 15
Slices	24	12
Acquisition time (min)	42 (37–44)	47

*NR* not reported

## Challenges and opportunities of renal MRI at 7 T

### RF Inhomogeneity

One of the main difficulties in abdominal imaging at 7 T results from the short RF wavelength, which negatively impacts transmit B_1_ homogeneity and efficiency while increasing local SAR concerns. Transmit B_1_ homogeneity and efficiency are interrelated, as increasing one often decreases the other when using standard static RF shimming techniques as discussed by Metzger et al. [14]. Inhomogeneous B_1_ can result in spatially dependent SNR and contrast [4]. Loss in transmit efficiency can limit the achievable B_1_^+^ needed for certain RF pulses such as those needed for refocusing and inversion. Finally, local SAR issues can greatly limit sequence and RF pulse design and timing in both cases. Even in low-flip-angle acquisitions, SAR limits become an issue as homogeneous solutions are often desired which, because of their decreased efficiency, require increased input power.

Although other solutions exist, most work on renal imaging at 7 T was performed using phase-only static RF shimming [14, 36, 42]. RF shimming consists of the use of a multiple-channel transmit coil, usually 8 channels or more, where each can be driven with unique amplitudes, phases, and/or pulse waveforms. These channels are connected to a transmit/receive or transceive array, positioned close to the subject, similar to traditional receive arrays, but where each element transmits RF signals as well. This multi-channel RF system provides the flexibility needed to address the B1 transmit inhomogeneity and efficiency issues when imaging the kidneys at 7 T.

**Fig. 15:**
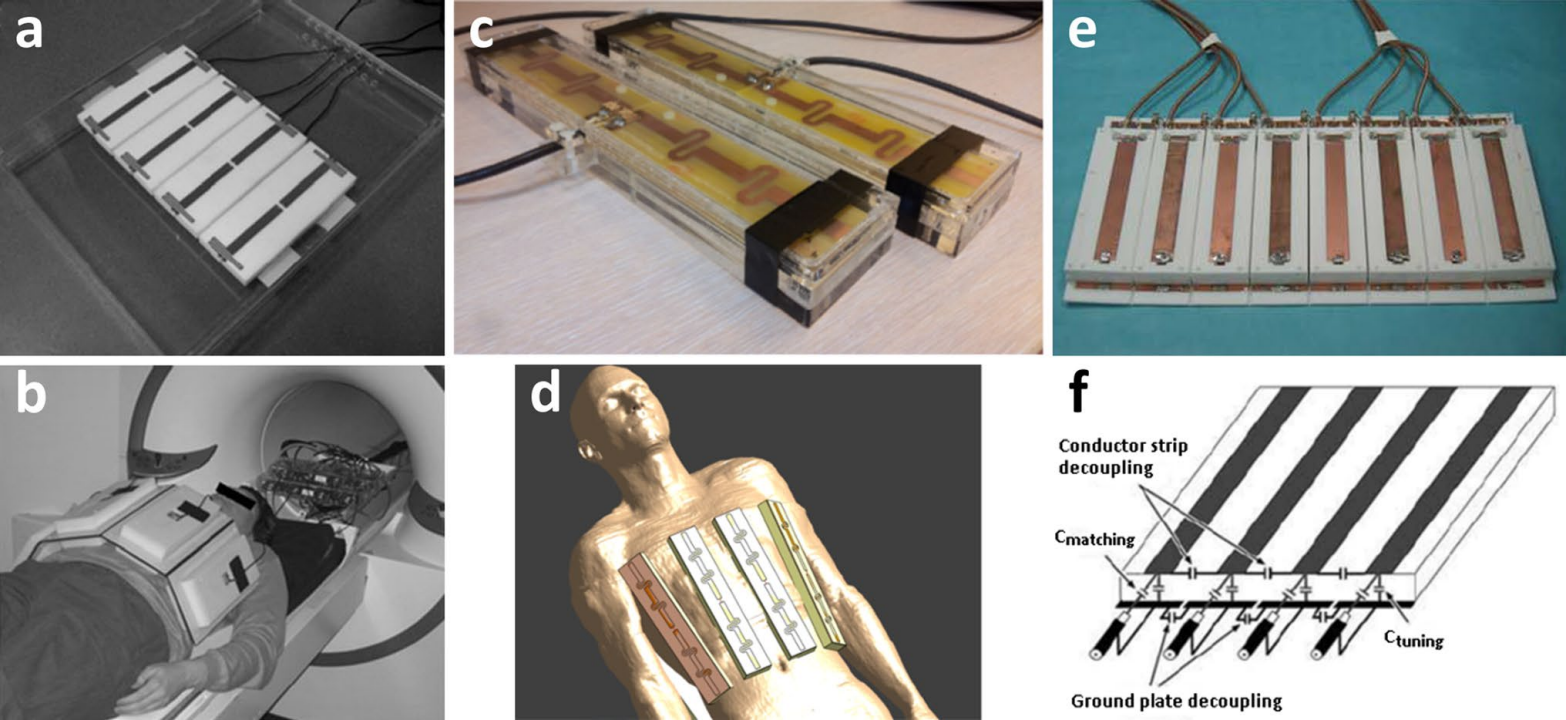
Different array coils and setups for renal 7 T imaging: **a** dorsal array of 8-channel array with microstrip meander elements used by Umutlu et al. (reprinted with permission from [36]). These elements consist of a central conductor over a ground plane, which are connected to each other by capacitors at both ends of the element while the element is fed in the centre. At each end of the element, extra inductance is added by a meander in the central conductor that effectively lowers SAR and reduces inter-element coupling; **b** setup of a; **c** two coil elements and **d** setup used by Hoogduin et al. [53]. The array consists of eight ‘fractionated dipole antennas’. Here, the legs of each dipole are divided into segments and the segments are interconnected by meanders (inductors). This element structure also reduces SAR levels and coupling in comparison to plain dipoles; **e** anterior array of 16-channel microstrip array used by Metzger et al. [47] (reprinted with permission from [55]). Here, a conductor is placed over a ground plate with capacitors connecting the two at both ends of the element (**f**). Capacitive coupling is used between the conductors and ground planes of adjacent elements to permit closer element spacing and higher element density. This element is driven from one side, over one of the connecting capacitors

Results presented in this paper have been acquired with three distinctly different coil arrays. Umutlu et al. used an 8 × 1 kW amplifier setup to drive an 8-channel array of microstrip elements (Fig. 15a, b) [36]. Hoogduin et al. used an 8 × 2 kW amplifier setup to drive an array of eight ‘fractionated dipole antennas’ (Fig. 15c, d) [53]. An extension of this array was recently presented where the dipole array is combined with a 16-element receive array, providing extra SNR [54]. Metzger et al. and Li et al. made use of 16 × 1 kW amplifiers to drive a 16-channel microstrip array (Fig. 15e, f) [55]. Technical details of these arrays are provided in Fig. 15. Sodium imaging results by Haneder et al. were acquired using a commercial 6-element spine array (Quality Electrodynamics, Mayfield Village, OH, USA) [51].

**Fig. 16:**
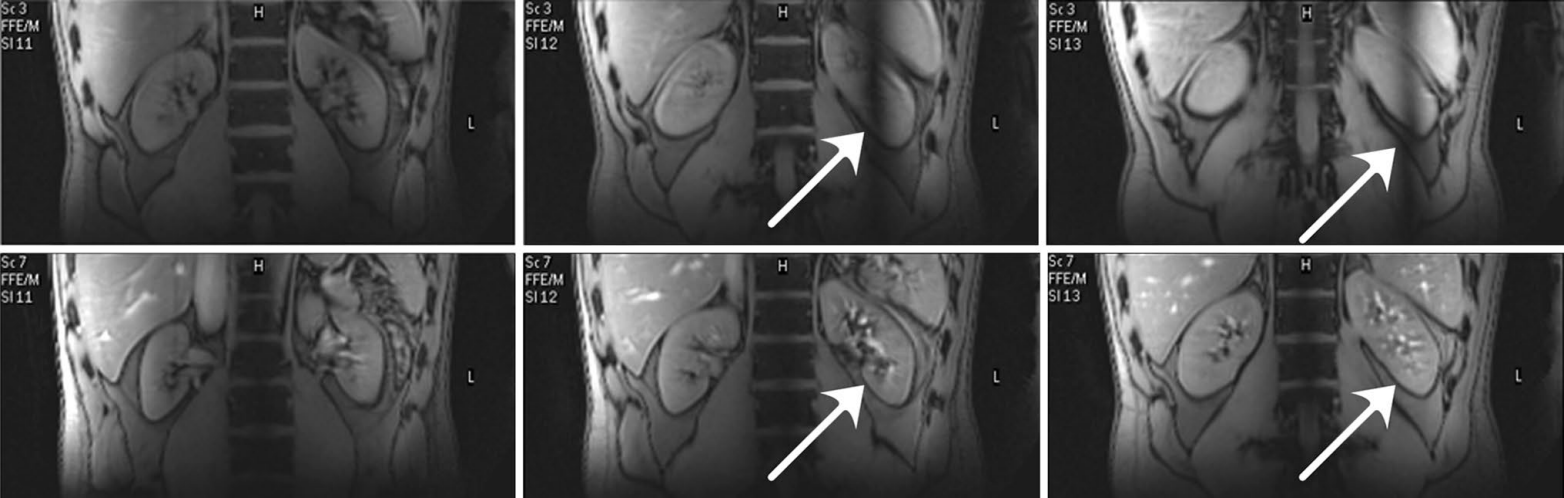
Survey pre (*upper row*) and post (*lower row*) RF shimming. Only phase shimming was performed. *Arrows* region of destructive interference in the *left kidney* in two of three images acquired before RF shimming, disappearing after shimming (Hoogduin et al. [37])

Using these multiple-channel coils, on each channel separately the phase of the RF signal can be adjusted to achieve B_1_^+^ homogeneity in a user-defined region of interest (ROI). In Fig. 16, an example of phase-only shimming is shown. To calculate the desired phase for each RF-channel, the magnitude and phase estimates of each transmit channels’ B_1_^+^ distribution are acquired. A relatively straightforward approach is to calculate the average B_1_^+^-phase over a user-defined ROI for each channel. Subsequently, this phase is subtracted from the transmit channel to obtain the same phase within the ROI for each channel [13]. Consequently, destructive interference of the fields within the ROI is minimized, maximizing the B_1_^+^ magnitude.

As demonstrated in the work by Metzger et al. [14], multiple B_1_^+^ shim solutions can also be used, each optimized for the specific requirement of RF pulses in the pulse sequence: homogeneity, efficiency or a trade-off between the two. These methods included a combination of both magnitude and phase shimming. This strategy was used in non-CE MRA of the renal arteries at 7 T [14]. Here, an adiabatic inversion RF pulse was applied during the MRA sequence to suppress background signal. This pulse is relatively insensitive to B_1_ inhomogeneity but requires a B_1_^+^ magnitude above a certain threshold. In contrast, a conventional excitation pulse requires a homogeneous B_1_^+^ field to assure a constant flip angle throughout the ROI. This is important since areas with a small B_1_^+^ field can mimic occluded vessels in MR angiograms. To avoid these artefacts, RF shimming can be performed twice, once to optimize B_1_^+^ efficiency and once to optimize homogeneity or a trade-off between the two. Complex B_1_^+^ maps were acquired for each channel using a quick algorithm proposed by Van de Moortele [56]. Optimization was performed by phase or magnitude and phase. Although varying both parameters potentially yields the best results in terms of B_1_^+^ homogeneity and magnitude, sometimes most of the RF power is distributed over a few channels. This yields the risk of exceeding the local specific absorption (SAR) limits. Therefore, phase-only optimization was preferentially used. However, when only small flip-angles are desired, the risk of exceeding SAR limits is smaller and magnitude can be included in the calculations.

Alternatives for RF shimming are, for example, transmit SENSE [57] and time interleaved acquisition of modes (TIAMO) [58]. In transmit SENSE, using the transmit profile of each coil and a user-defined gradient trajectory, a unique RF-waveform is calculated for each transmit channel. Consequently, transmit SENSE requires hardware that is capable of producing an arbitrary RF waveform for individual channels. In TIAMO, two acquisitions are performed with different excitation modes of the RF pulse—for example, a 45° phase increment along RF coils followed by a 90° increment. Using parallel imaging techniques, the two acquisitions can be acquired in the same time as needed for a single image [58]. Using GRAPPA, the final image is reconstructed from the two acquisitions.

### SAR limitations

MR imaging at 7 T is associated with higher SAR levels. More power is needed since the penetration depth becomes smaller. This is because the electric fields that are induced are larger, which also results in higher SAR levels. However, at 7 T, local transmit coil arrays are being used. These arrays are positioned much closer to the patient and are therefore much more efficient. The local transmit array of dipole antennas only needs 8 × 2 kW to reach the same B_1_ levels for which a 3T body coil requires 2 × 16 kW. So although 7 T B_0_ fields are associated with higher SAR levels, this does not apply for global SAR since much more efficient local transmit arrays are being used at 7 T. Nevertheless, to ensure that global SAR does not exceed limits, multiple groups implemented real-time power monitoring [14, 36].

**Fig. 17:**
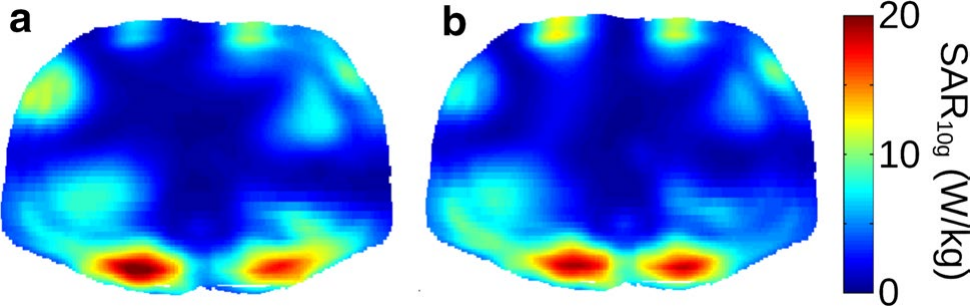
Local SAR distributions (10 g averaged) for two phase-amplitude settings that are both designed for constructive interference of B1 in the kidneys. Distributions are in the transverse plane crossing the maximum value in the distribution. Values are for 8 × 800 W, with 1 % duty cycle (Raaijmakers, unpublished results)

However, the use of local transmit arrays can lead to large variations in energy deposition [14]. Therefore, local SAR also has to be taken into account. The IEC guidelines [59] prescribe a limit for the maximum allowed 10 g averaged local SAR value anywhere in the exposed region (peak local SAR). The exact limit depends on the body part and the surveillance mode but ranges from 10 to 40 W/kg. Unlike global SAR, local SAR cannot be measured; it has to be calculated from numerically simulated field distributions and the applied phase and amplitude settings of the transmit array. An example of such simulated local SAR distributions is provided in Fig. 17 for two different shim settings, both optimized for kidney imaging. The peak local SAR in these simulated distributions dictates the minimum repetition time, taking into account an uncertainty margin because of inter-subject variability [60]. Thus, the peak local SAR limits are what constrain 7 T kidney imaging.

An important method with which to reduce SAR is parallel imaging. Since fewer phase encoding steps are required, less RF-pulses are needed and acquisition time is decreased. However, it does reduce SNR—so in principle it enables us to acquire the same SNR at the same SAR in less time on high fields as compared to low fields [61]. Other options to reduce SAR include reducing echo train length or increasing repetition time, but this directly influences acquisition time [12]. Simply decreasing the flip angle has a significant influence on SAR, since SAR changes quadratically with flip angle for a given RF pulse [12]. However, a decreased flip angle also influences image contrast. Alternatively, the power required by the RF pulse can be minimized, for example, using ‘variable-rate selective excitation’ (VERSE) [62] or, when adiabatic pulses are desired, using ‘gradient-modulated offset-independent adiabaticity’ (GOIA) [63].

TSE sequences have high energy deposition due to the refocusing 180° pulse trains used. It is possible to reduce the flip angles in this pulse train using ‘transition between pseudo steady states’ (TRAPS) [64] or using hyperechoes [65]. Both techniques employ the fact that image contrast is stored in the centre of k-space. While the centre of k-space should be sampled with a sufficiently large flip angle, smaller flip angles can be used for the periphery.

## Conclusion and discussion

Although the feasibility of numerous sequences on 7 T in the kidney has been demonstrated, there are still a number of difficulties that need to be overcome. SAR limitations seem to impose most restrictions on sequence optimization. Techniques as parallel imaging and the use of hyperechoes have partially overcome these problems, but come at the cost of SNR. RF inhomogeneity can be successfully dealt with using RF shimming, and more advanced techniques are being developed, potentially leading to superior B_1_^+^ homogeneity [57, 58].

Anatomical imaging seems to suffer most from SAR constraints and to a lesser degree from RF inhomogeneity. In *T*_1_-weighted sequences, resolutions of about 1 mm are reached within about half a minute acquisition time. In conventional clinical imaging at 3 T, results are comparable in terms of spatial resolution and scan duration [66]. *T*_2_-weighted imaging, however, is strongly impaired by SAR constraints, resulting in artefacts or increased imaging times, although spatial resolution could be slightly improved.

Excellent image quality of the renal vascular system at 7 T using non-CE sequences suggest a bright future for this technique. Although the feasibility was shown by two groups [14, 40], no direct comparison between non-CE and CE MRA is available. Comparison of the CNRs measured for TOF MRA and CE MRA yields a two-fold increase in CNR for CE MRA (28 versus a range of 45 to 60), but quality scores barely differed (4.7 versus 4.8) [40, 41]. In addition, no information on dosage and timing of contrast agent administration is yet available and no pathology was studied.

Benefits of functional renal MRI at 7 T potentially are large and address the important clinical need for techniques that can better assess local and split renal function. Theoretically, both techniques currently applied in the kidneys profit from the high field strength. BOLD MRI benefits from increased sensitivity to susceptibility effects, while ASL utilizes the increase in T_1_ relaxation times. Promising results are obtained in sodium imaging. Since the RF wavelength related to the sodium nucleus is about 45 cm in tissue, transmit inhomogeneity is not an issue. Thanks to the increased SNR at 7 T compared to 3 T, resolution could be increased by 36 % [51].

In conclusion, although numerous challenges still have to be overcome, the future of renal 7 T MRI is promising, especially for functional imaging techniques.

## References

[1] Robitaille PML, Abduljalil AM, Kangarlu A, Zhang X, Yu Y, Burgess R, Bair S, Noa P, Yang L, Zhu H, Palmer B, Jiang Z, Chakeres DM, Spigos D (1998). Human magnetic resonance imaging at 8 T. NMR Biomed.

[2] Ashman CJ, Farooki S, Abduljalil AM, Chakeres DW (2002). In vivo high resolution coronal MRI of the wrist at 8.0 tesla. J Comput Assist Tomogr.

[3] Metzger GJ, Van de Moortele PF, Snyder CJ, Vaughan JT, Ugurbil K (2007) Local B1 Shimming for Imaging the Prostate at 7 Tesla. In: Proceedings of the 15th scientific meeting, International Society for Magnetic Resonance in Medicine, Berlin, p 799

[4] Vaughan JT, Snyder CJ, Delabarre LJ, Bolan PJ, Tian J, Bolinger L, Adriany G, Andersen P, Strupp J, Ugurbil K (2009). Whole-body imaging at 7 T: preliminary results. Magn Reson Med.

[5] Snyder CJ, DelaBarre L, Metzger GJ, van de Moortele PF, Akgun C, Ugurbil K, Vaughan JT (2009). Initial results of cardiac imaging at 7 Tesla. Magn Reson Med.

[6] Dieringer MA, Renz W, Lindel T, Seifert F, Frauenrath T, von Knobelsdorff-Brenkenhoff F, Waiczies H, Hoffmann W, Rieger J, Pfeiffer H, Ittermann B, Schulz-Menger J, Niendorf T (2011). Design and application of a four-channel transmit/receive surface coil for functional cardiac imaging at 7 T. J Magn Reson Imaging.

[7] Moser E, Stahlberg F, Ladd ME, Trattnig S (2012). 7-T MR-from research to clinical applications?. NMR Biomed.

[8] Li X, Bolan PJ, Ugurbil K, Metzger GJ (2015). Measuring renal tissue relaxation times at 7 T. NMR Biomed.

[9] Shen Y, Goerner FL, Snyder C, Morelli JN, Hao D, Hu D, Li X, Runge VM (2015). T1 relaxivities of gadolinium-based magnetic resonance contrast agents in human whole blood at 1.5, 3, and 7 T. Invest Radiol.

[10] Kalavagunta C, Michaeli S, Metzger GJ (2014). In vitro Gd-DTPA relaxometry studies in oxygenated venous human blood and aqueous solution at 3 and 7 T. Contrast Media Mol Imaging.

[11] Noebauer-Huhmann IM, Szomolanyi P, Juras V, Kraff O, Ladd ME, Trattnig S (2010). Gadolinium-based magnetic resonance contrast agents at 7 Tesla: in vitro *T*1 relaxivities in human blood plasma. Invest Radiol.

[12] McRobbie DW, Moore EA, Graves MJ, Prince MR (2015). MRI from picture to proton.

[13] Metzger GJ, Snyder C, Akgun C, Vaughan T, Ugurbil K, Van De Moortele PF (2008). Local B1+ shimming for prostate imaging with transceiver arrays at 7 T based on subject-dependent transmit phase measurements. Magn Reson Med.

[14] Metzger GJ, Auerbach EJ, Akgun C, Simonson J, Bi X, Uǧurbil K, De Van, Moortele PF (2013). Dynamically applied B1+ shimming solutions for non-contrast enhanced renal angiography at 7.0 tesla. Magn Reson Med.

[15] Moore KL, Dalley AF, Agur AMR (2010). Clinically oriented anatomy.

[16] De Jong PE, Koomans HA, Weening JJ (2011). Klinische nefrologie.

[17] El-Reshaid W, Abdul-Fattah H (2014). Sonographic assessment of renal size in healthy adults. Med Princ Pract.

[18] Rhoades RA, Bell DR (2013). Medical physiology: principles for clinical medicine.

[19] el-Galley RE, Keane TE (2000). Embryology, anatomy, and surgical applications of the kidney and ureter. Surg Clin North Am.

[20] Zollner FG, Konstandin S, Lommen J, Budjan J, Schoenberg SO, Schad LR, Haneder S (2016). Quantitative sodium MRI of kidney. NMR Biomed.

[21] Brezis M, Rosen S (1995). Hypoxia of the renal medulla—its implications for disease. N Engl J Med.

[22] Prasad PV, Edelman RR, Epstein FH (1996). Noninvasive evaluation of intrarenal oxygenation with BOLD MRI. Circulation.

[23] Brachemi S, Bollee G (2014). Renal biopsy practice: what is the gold standard?. World J Nephrol.

[24] Tendera M, Aboyans V, Bartelink ML, Baumgartner I, Clement D, Collet JP, Cremonesi A, De Carlo M, Erbel R, Fowkes FG, Heras M, Kownator S, Minar E, Ostergren J, Poldermans D, Riambau V, Roffi M, Rother J, Sievert H, van Sambeek M, Zeller T (2011). ESC Guidelines on the diagnosis and treatment of peripheral artery diseases: document covering atherosclerotic disease of extracranial carotid and vertebral, mesenteric, renal, upper and lower extremity arteries: the Task Force on the Diagnosis and Treatment of Peripheral Artery Diseases of the European Society of Cardiology (ESC). Eur Heart J.

[25] Ljungberg B, Bensalah K, Bex A et al (2015) Guidelines on renal cell carcinoma. European Association of Urology. http://uroweb.org/wp-content/uploads/EAU-Guidelines-Renal-Cell-Cancer-2015-v2.pdf. Accessed 16 Dec 2015

[26] Ramamurthy NK, Moosavi B, McInnes MD, Flood TA, Schieda N (2015). Multiparametric MRI of solid renal masses: pearls and pitfalls. Clin Radiol.

[27] Wang YT, Li YC, Yin LL, Pu H, Chen JY (2015). Functional assessment of transplanted kidneys with magnetic resonance imaging. World J Radiol.

[28] Gloviczki ML, Saad A, Textor SC (2013). Blood oxygen level-dependent (BOLD) MRI analysis in atherosclerotic renal artery stenosis. Curr Opin Nephrol Hypertens.

[29] Lim SW, Chrysochou C, Buckley DL, Kalra PA, Sourbron SP (2013). Prediction and assessment of responses to renal artery revascularization with dynamic contrast-enhanced magnetic resonance imaging: a pilot study. Am J Physiol Renal Physiol.

[30] Dong J, Yang L, Su T, Yang X, Chen B, Zhang J, Wang X, Jiang X (2013). Quantitative assessment of acute kidney injury by noninvasive arterial spin labeling perfusion MRI: a pilot study. Sci China Life Sci.

[31] Michaely HJ, Metzger L, Haneder S, Hansmann J, Schoenberg SO, Attenberger UI (2012). Renal BOLD-MRI does not reflect renal function in chronic kidney disease. Kidney Int.

[32] Prasad PV, Thacker J, Li LP, Haque M, Li W, Koenigs H, Zhou Y, Sprague SM (2015). Multi-parametric evaluation of chronic kidney disease by MRI: a preliminary cross-sectional study. PLoS One.

[33] Zeng M, Cheng Y, Zhao B (2015). Measurement of single-kidney glomerular filtration function from magnetic resonance perfusion renography. Eur J Radiol.

[34] Vink EE, de Jager RL, Blankestijn PJ (2013). Sympathetic hyperactivity in chronic kidney disease: pathophysiology and (new) treatment options. Curr Hypertens Rep.

[35] Niendorf T, Pohlmann A, Arakelyan K, Flemming B, Cantow K, Hentschel J, Grosenick D, Ladwig M, Reimann H, Klix S, Waiczies S, Seeliger E (2015). How bold is blood oxygenation level-dependent (BOLD) magnetic resonance imaging of the kidney? Opportunities, challenges and future directions. Acta Physiol (Oxf).

[36] Umutlu L, Orzada S, Kinner S, Maderwald S, Brote I, Bitz AK, Kraff O, Ladd SC, Antoch G, Ladd ME, Quick HH, Lauenstein TC (2011). Renal imaging at 7 Tesla: preliminary results. Eur Radiol.

[37] Hoogduin H, Raaijmakers A, Visser F, Luijten P (2014) Initial experience with BOLD imaging of the kidneys at 7 T. In: Proceedings of the 22nd scientific meeting, International Society for Magnetic Resonance in Medicine, Milan, p 456

[38] Grinstead JW, Rooney W, Laub G (2010) The origins of bright blood MPRAGE at 7 Tesla and a simultaneous method for T1 imaging and non-contrast MRA. In: Proceedings of the 18th scientific meeting, International Society for Magnetic Resonance in Medicine, Stockholm, p 1429

[39] Umutlu L, Kraff O, Orzada S, Fischer A, Kinner S, Maderwald S, Antoch G, Quick HH, Forsting M, Ladd ME, Lauenstein TC (2011). Dynamic contrast-enhanced renal MRI at 7 Tesla preliminary results. Invest Radiol.

[40] Umutlu L, Maderwald S, Kraff O, Kinner S, Schaefer LC, Wrede K, Antoch G, Forsting M, Ladd ME, Lauenstein TC, Quick HH (2012). New look at renal vasculature: 7 tesla nonenhanced T1-weighted FLASH imaging. J Magn Reson Imaging.

[41] Umutlu L, Maderwald S, Kinner S, Kraff O, Bitz AK, Orzada S, Johst S, Wrede K, Forsting M, Ladd ME, Lauenstein TC, Quick HH (2013). First-pass contrast-enhanced renal MRA at 7 Tesla: initial results. Eur Radiol.

[42] Brinkmann I, Darji N, Speck O, Bock M (2014) BOLD MRI of the Kidneys under water loading at 7 Tesla using parallel transmission and RF shimming of individual slices. In: Proceedings of the 22nd scientific meeting, International Society for Magnetic Resonance in Medicine, Milan, p 3557

[43] Ebrahimi B, Gloviczki M, Woollard JR, Crane JA, Textor SC, Lerman LO (2012). Compartmental analysis of renal BOLD MRI data: introduction and validation. Invest Radiol.

[44] Vink EE, de Boer A, Hoogduin HJ, Voskuil M, Leiner T, Bots ML, Joles JA, Blankestijn PJ (2015). Renal BOLD-MRI relates to kidney function and activity of the renin-angiotensin-aldosterone system in hypertensive patients. J Hypertens.

[45] Wong EC (2014). An introduction to ASL labeling techniques. J Magn Reson Imaging.

[46] Zhang X, Petersen ET, Ghariq E, De Vis JB, Webb AG, Teeuwisse WM, Hendrikse J, van Osch MJ (2013). In vivo blood T(1) measurements at 1.5 T, 3 T, and 7 T. Magn Reson Med.

[47] Snyder CJ, Delabarre L, Moeller S, Tian J, Akgun C, Van de Moortele PF, Bolan PJ, Ugurbil K, Vaughan JT, Metzger GJ (2012). Comparison between eight- and sixteen-channel TEM transceive arrays for body imaging at 7 T. Magn Reson Med.

[48] Li X, Ugurbil K, Metzger G (2013) Theoretical evaluation of ultrahigh field benefits to non-contrast enhanced renal perfusion imaging using FAIR-EPI. In: Proceedings of the 21th scientific meeting, International Society for Magnetic Resonance in Medicine, Salt Lake City, p 1540

[49] Li X, Snyder C, Van de Moortele PF, Ugurbil K, Metzger G (2012) Non-contrast enhanced human renal perfusion imaging using arterial spin labeling at 7 T: initial experience. In: Proceedings of the 20th scientific meeting, International Society for Magnetic Resonance in Medicine, Melbourne, p 1310

[50] Li X, Snyder C, Van de Moortele PF, Ugurbil K, Metzger G (2013) Feasibility of single breath-hold renal perfusion imaging at 7 T. In: Proceedings of the 21st scientific meeting, International Society for Magnetic Resonance in Medicine, Salt Lake City, p 30

[51] Haneder S, Juras V, Michaely HJ, Deligianni X, Bieri O, Schoenberg SO, Trattnig S, Zbýň S (2014). In vivo sodium (23Na) imaging of the human kidneys at 7 T: preliminary results. Eur Radiol.

[52] Haneder S, Konstandin S, Morelli JN, Nagel AM, Zoellner FG, Schad LR, Schoenberg SO, Michaely HJ (2011). Quantitative and qualitative 23 Na MR imaging of the human kidneys at 3 T: before and after a water load. Radiology.

[53] Raaijmakers AJ, Italiaander M, Voogt IJ, Luijten PR, Hoogduin JM, Klomp DW, van den Berg CA (2015). The fractionated dipole antenna: a new antenna for body imaging at 7 Tesla. Magn Reson Med.

[54] Voogt IJ, Klomp D, Hoogduin H, Luttje MP, Luijten P, van den Berg CAT, Raaijmakers A (2015) Combined 8-Channel Transceiver Fractionated Dipole Antenna Array with a 16-Channel Loop Coil Receive Array for Body Imaging at 7 Tesla. In: Proceedings of the 23rd scientific meeting, International Society for Magnetic Resonance in Medicine, Toronto, p 631

[55] Metzger GJ, van de Moortele PF, Akgun C, Snyder CJ, Moeller S, Strupp J, Andersen P, Shrivastava D, Vaughan T, Ugurbil K, Adriany G (2010). Performance of external and internal coil configurations for prostate investigations at 7 T. Magn Reson Med.

[56] Van de Moortele PF, Ugurbil K (2009) Very fast multi channel B1 calibration at high field in the small flip angle regime. In: Proceedings of the 17 Th scientific meeting, International Society for Magnetic Resonance in Medicine, Honolulu, p 367

[57] Katscher U, Börnert P, Leussler C, Van den Brink JS (2003). Transmit SENSE. Magn Reson Med.

[58] Orzada S, Maderwald S, Poser BA, Bitz AK, Quick HH, Ladd ME (2010). RF excitation using time interleaved acquisition of modes (TIAMO) to address B1 inhomogeneity in high-field MRI. Magn Reson Med.

[59] IEC (2002). Medical electrical equipment—part 2–33: particular requirements for the basic safety and essential performance of magnetic resonance equipment for medical diagnosis.

[60] Ipek O, Raaijmakers AJ, Lagendijk JJ, Luijten PR, van den Berg CA (2014). Intersubject local SAR variation for 7 T prostate MR imaging with an eight-channel single-side adapted dipole antenna array. Magn Reson Med.

[61] Schmitz BL, Aschoff AJ, Hoffmann MH, Gron G (2005). Advantages and pitfalls in 3T MR brain imaging: a pictorial review. AJNR Am J Neuroradiol.

[62] Conolly S, Nishimura D, Macovski A, Glover G (1988). Variable-rate selective excitation. J Magn Reson 1969.

[63] Tannus A, Garwood M (1997). Adiabatic pulses. NMR Biomed.

[64] Hennig J, Weigel M, Scheffler K (2003). Multiecho sequences with variable refocusing flip angles: optimization of signal behavior using smooth transitions between pseudo steady states (TRAPS). Magn Reson Med.

[65] Hennig J, Scheffler K (2001). Hyperechoes. Magn Reson Med.

[66] De Keyzer F, Thoeny HC (2010). Renal and perfusion imaging at 3 T. Top Magn Reson Imaging.

